# Phenotypic plasticity in normal breast derived epithelial cells

**DOI:** 10.1186/1471-2121-15-20

**Published:** 2014-06-10

**Authors:** Candice AM Sauder, Jillian E Koziel, MiRan Choi, Melanie J Fox, Brenda R Grimes, Sunil Badve, Rachel J Blosser, Milan Radovich, Christina C Lam, Melville B Vaughan, Brittney-Shea Herbert, Susan E Clare

**Affiliations:** 1 Department of Surgery, Indiana University School of Medicine, 980 W. Walnut Street, Indianapolis, IN 46202, USA; 2 Department of Medical and Molecular Genetics, Indiana University School of Medicine, 975 W. Walnut Street, Indianapolis, IN 46202, USA; 3 Department of Surgery, Feinberg School of Medicine, Northwestern University, 303 E. Superior Street, Chicago, IL 60611, USA; 4 Department of Pathology, Indiana University School of Medicine, 350 West 11th Street, Indianapolis, IN 46202, USA; 5 Department of Biology, University of Central Oklahoma, 100 North University Drive, Edmond, OK 73034, USA

**Keywords:** Plasticity, Breast, Metaplasia, Squamous, Basal, Embryonic, Epithelium

## Abstract

**Background:**

Normal, healthy human breast tissue from a variety of volunteer donors has become available for research thanks to the establishment of the Susan G. Komen for the Cure® Tissue Bank at the IU Simon Cancer Center (KTB). Multiple epithelial (K-HME) and stromal cells (K-HMS) were established from the donated tissue. Explant culture was utilized to isolate the cells from pieces of breast tissue. Selective media and trypsinization were employed to select either epithelial cells or stromal cells. The primary, non-transformed epithelial cells, the focus of this study, were characterized by immunohistochemistry, flow cytometry, and *in vitro* cell culture.

**Results:**

All of the primary, non-transformed epithelial cells tested have the ability to differentiate *in vitro* into a variety of cell types when plated in or on biologic matrices. Cells identified include stratified squamous epithelial, osteoclasts, chondrocytes, adipocytes, neural progenitors/neurons, immature muscle and melanocytes. The cells also express markers of embryonic stem cells.

**Conclusions:**

The cell culture conditions employed select an epithelial cell that is pluri/multipotent. The plasticity of the epithelial cells developed mimics that seen in metaplastic carcinoma of the breast (MCB), a subtype of triple negative breast cancer; and may provide clues to the origin of this particularly aggressive type of breast cancer. The KTB is a unique biorepository, and the normal breast epithelial cells isolated from donated tissue have significant potential as new research tools.

## Background

**Figure 1:**
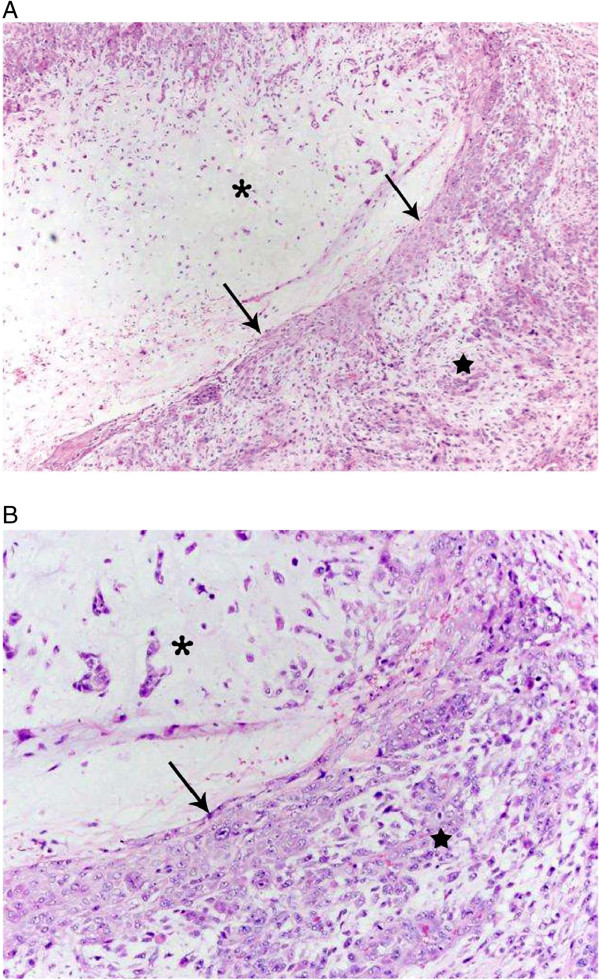
**Hematoxylin and eosin stained section of a metaplastic carcinoma of the breast.** Asterisk indicates chondrocytic differentiation, arrows mark squamous differentiation and star is in the middle of spindle cell differentiation. **A**. Low-magnification view, 4x. **B**. Higher magnification of the section showing all three cell types.

Metaplasia is the appearance of a tissue type foreign to the organ it arises in and results from a reprogramming of stem cells that reside in normal tissues [1]. In the human breast, it is observed in both benign and malignant lesions. The malignant version, metaplastic carcinoma of the breast (MCB), encompasses squamous carcinoma, squamous carcinoma with spindle cell metaplasia, carcinoma with chondroid differentiation, carcinoma with osseous differentiation, and adenocarcinoma with spindle cell differentiation [2]. Not infrequently, the metaplasia is not limited to one cell type (Figure 1). Gwin *et al.* studied twenty-one infiltrating duct carcinomas with chondroid differentiation; fully a third also exhibited squamous metaplasia [3]. An interesting observation is that cartilage and bone formation are relatively frequent occurrences in the mammary tumors of cats and dogs [4]. Canine tumors containing epithelial and mesenchymal components are hypothesized to arise from stem cells based on the fact that the two components are monoclonal [5,6]. Bone and cartilage are also observed in pleomorphic adenoma of the salivary glands and rarely in Calcifying Epithelioma of Malherbe [2]. Metaplasia also arises in the context of chronic inflammation; for example, *H. pylori* infection of the gastric mucosa induces intestinal metaplasia. This conversion is hypothesized to be the result of a change in the expression of one or several transcription factors in the adult stem cell [7].

The Susan G. Komen for the Cure® Tissue Bank at the IU Simon Cancer Center is a biorepository established expressly for the acquisition of normal, i.e. healthy, breast tissue from volunteer donors [8,9]. To increase the availability of a prohibitively limited resource, epithelial (K-HME) and stromal cells (K-HMS) were established from the donated tissue. The primary, non-transformed epithelial cells, the focus of this study, were characterized by immunohistochemistry, flow cytometry, and *in vitro* cell culture. During this process it has become apparent that all of the epithelial cells tested have the ability to differentiate *in vitro* into a variety of cell types when plated in or on biologic matrices. Classic germ layer theory posits that some of these cell types have their origin in the ectoderm but others are derived from the mesoderm or neural crest. However, here is a growing body of evidence to suggest that explant culture conditions, such as were utilized to isolate these cells, select cells that are multipotent [10-14]. The plasticity of the epithelial cells developed by KTB mimics that seen in MCB and it is tempting to postulate that these tumors arise from similar multipotent or plastic cells as described in the present study.

## Methods

All studies were approved by the Indiana University Institutional Review Board (IRB-04; protocol number 0709–17; and NS1006-04). All research was carried out in compliance with the Helsinki Declaration.

### Experimental design

The original purpose of this study was the characterization of the epithelial cells derived from the donated normal breast tissue. Initial studies included immunohistochemistry to determine the expression of epithelial cytokeratins, myoepithelial markers and hormone receptors; and ploidy analysis. The first evidence of plasticity was observed in the Matrigel® cultures. This observation shifted the focus of this study to an assay of the epithelial plasticity. As Matrigel® is a mixture of collagen, laminin and fibronectin, cells were grown on each of these surfaces to determine if any one of these proteins was responsible for the transformed phenotype. Preliminary cell type identifications were made based upon shape, size and intracellular components on phase contrast microscopy. Colorimetric assays and immunohistochemistry were utilized to provide additional evidence as to the cells’ identities. Alcian blue is routinely used in pathology laboratories to identify cartilage and nestin expression is a marker of neural stem cells. However, as both have been noted in normal breast epithelium [15,16], additional makers of chondrocytic and neural differentiation that are specific to the cell type were assayed.

### Cell culture

After obtaining informed consent, a 10 gauge core needle was used to obtain breast tissue (<100 mg) from 39 healthy female volunteers with no history of breast disease (see Additional file 1: Table S1 for age, race, and Gail risk score) [8]. The tissue was immediately homogenized, digested with collagenase and hyaluronidase, and cultured using selective media and trypsinization to differentiate the epithelial cells from stromal cells as previously described [17-21]. The KTB HME cells (K-HME; K-HME 490, K-HME 509, K-HME 538, K-HME 496, and K-HME 511) were obtained from frozen stocks, thawed, and suspended in WIT-P media (Stemgen, San Diego, CA, USA) unless otherwise stated (see Additional file 2: Methods) then plated onto Primaria-coated T-25 flasks (BD Bioscience, San Jose, CA, USA). The majority of experiments used cells prior to passage four; no follow-up experiments utilized cells beyond passage 14. Cells were cultured at 37°C, in an environment of 95% relative humidity and 5% CO_2_.

### Ploidy status

Fluorescence in situ hybridization (FISH) with centromere probes from chromosome X (CEPX) and chromosome 17 (CEP17) were performed as described by Grimes and colleagues [22].

### 3-D Matrigel® culture

125,000 K-HME cells were grown in the middle of a Matrigel® (BD Bioscience, San Jose, CA, USA) sandwich culture in a 24-well plate. After approximately 10 days, the cultures were encapsulated in HistoGel™ (Richard-Allen Scientific, Kalamazoo, MI, USA), formalin-fixed and paraffin-embedded. Immunohistochemistry (IHC) was performed by the IUH Pathology Laboratory using the Dako AutostainerPlus (Dako, Carpinteria, CA, USA) and the protocols given in Additional file 1: Table S2. The primary antibody was eliminated to prepare the negative controls of all immunohistochemistry reactions.

### Differentiation analyses of cultures grown on coated surfaces

#### Chondrocytic differentiation

6-well plates coated with Type IV Collagen, Laminin, Fibronectin, and Primaria™ surface treatment were obtained from BD Biosciences (San Jose, CA, USA). 6.5 × 10^5^ K-HME cells were pipetted onto each of the coated surfaces of the culture plates. Cells were incubated for 10–12 days with a change of media every other day. The Alcian blue pH 2.5 Stain Kit (Artisan™, Dako, Carpinteria, CA, USA) was used per manufacturer’s instructions.

Two wells of a 4-well culture slide (Lab-Tek, Scotts Valley, CA, USA, #154526) were coated with collagen (Stem Cell Technologies, Vancouver, BC, Canada; #04902) and 2.5 × 10^4^ cells were plated per well. The cells were grown on the surface of the slide or on the collagen in WIT-P media for 6 days. Cells were fixed in 10% buffered formalin. Anti-Collagen II and X IHC was carried out as given in Additional file 1: Table S2.

#### Osteocytic differentiation

Cell culture plates, cell number and incubation duration are as given for chondrocytic differentiation. The TRACP & ALP double-stain kit (Takara*,* Shiga, Japan) was used per manufacturer’s instructions.

#### Adipocytic differentiation

Cell culture plates, cell number and incubation duration are as given for chondrocytic differentiation. Cells were fixed in paraformaldehyde (4% paraformaldehyde, 0.15% picric acid in PBS) for 1 hour at RT and then incubated with Oil Red O (Sigma-Aldrich, St. Louis, MO, USA) for 1 hour at 37°C.

#### Neural differentiation

Human Type IV Collagen was obtained from BD Biosciences (San Jose, CA, USA) and diluted 1:5 in 10 mM acetic acid). 125 μl of the collagen solution was used to coat the bottom of each well of the 8-well culture slides (BD Falcon, Franklin Lakes, NJ, USA; cat. no. 354118). Human nestin monoclonal antibody (Clone 196908, MAB 1259), neuron-specific beta-III tubulin monoclonal antibody (Clone TuJ-1, MAB 1195), and NorthernLights™ 557-conjugated sheep polyclonal anti-human GFAP (NL2594R) were obtained from R&D Systems, Minneapolis, MN, USA. Human nestin and neuron-specific beta-III tubulin antibody solutions were diluted 1:100 in blocking buffer (BB) and incubated with the cells overnight at 4°C. Anti-human GFAP was diluted 1:10 in BB and incubated with the cells for 3 hours in the dark at RT. Cells and primary antibody were incubated with secondary antibody (1:200 in BB; Northern Lights™ fluorescent secondary antibody NL*-*557 anti*-*Mouse IgG (NL007); R&D Systems, Minneapolis, MN, USA) for 1 hour in the dark at RT. They then were incubated for 5 minutes with 300 nM DAPI (Molecular Probes, Grand Island, NY, USA) in the dark. They were stored at 4°C until microscopy.

Two wells of a 4-well culture slide (Lab-Tek, Scotts Valley, CA, USA, #154526) were coated with collagen (Stem Cell Technologies, Vancouver, BC, Canada; #04902) and 2.5 × 10^4^ cells were plated per well. The cells were grown on the surface of the slide or on the collagen in WIT-P media for 6 days. Cells were fixed in 10% buffered formalin. Neu-N IHC was carried out as given in Additional file 1: Table S2.

#### Melanocytic differentiation

Two wells of a 4-well culture slide (BD Falcon, Franklin Lakes, NJ, USA; #354104) were coated with collagen (Stem Cell Technologies, Vancouver, BC, Canada; #04902) and 3 × 10^4^ cells were plated per well. The cells were grown on the surface of the slide or on the collagen, and in either Melanocyte Growth Medium (ZenBio, Inc., Durham, NC, USA) or WIT-P. After 3 days, the cells were fixed in 4% paraformaldehyde. Anti-MART-1 IHC was carried out as given in Additional file 1: Table S2.

#### Organotypic culture

Normal human dermal fibroblasts and Type 1 Collagen (Becton Dickinson, Franklin Lakes, NJ, USA) were mixed, polymerized and cultured as previously described [23] with modifications. After allowing 2 days for the fibroblasts to contract and reorganize the tissue, K-HME cells were plated on the upper surface at a concentration to provide a confluent monolayer of cells (200,000 cells per cm^2^). Culture continued for 2 days submerged in keratinocyte medium [24] with 20 μg/ml ascorbic acid (Sigma-Aldrich, St. Louis, MO, USA). For air/liquid interface culture, tissues were transferred to clear Transwell 6-well inserts (Corning Costar, Tewksbury, MA, USA) and placed into deep-well dishes (BioCoat, Becton Dickinson, Franklin Lakes, NJ, USA) for the remainder of the culture period. 16 hours prior to harvest, cultures were treated with 10 μM 5-ethynyl-2′-deoxyuridine (EdU; Invitrogen, Carlsbad, CA, USA) to label proliferating cells. Samples were harvested at 1, 3, and 7 days then prepared for frozen sections.

### Immunohistochemistry

5 × 10^5^ cells in WIT-P media were plated per well of a chamber slide (BD Falcon, San Jose, CA, USA) and the cells incubated overnight. Cells were fixed using 4% paraformadehyde, 0.15% picric acid in 1 × PBS for 20 minutes at RT. OCT4, NANOG and MyoD immunohistochemistry were performed per the protocols given in Additional file 1: Table S2. EGFR IHC was performed using the EGFR (Dako) RTU primary antibody and the EGFR pharmDX Kit (Dako), which was used according to the manufacturer’s instructions.

### Immunofluorescence

Cells grown on chamber slides (BD Falcon, Franklin Lakes, NJ, USA) were fixed with 4% paraformaldehyde (10 min.) and permeabilized with 0.1% Triton X-100 (10 min.) before blocking with 3% BSA in 1 × PBS (1 hr). Primary antibodies (CK 5/6, CK 8/18, CK 14, CK 19, EMA, SMA, Thermo Fisher Scientific, Kalamazoo, MI, USA; p63, Sigma-Aldrich, St. Louis, MO, USA; vimentin, Cell Signaling, Danvers, MA, USA) were diluted 1:100 in 1% BSA in 1 × PBS and incubated with the cells for 2 hrs. Binding of a 1:600 dilution of appropriate Alexa-Fluor 468 anti-mouse IgG or Alexa-Fluor 588 anti-rabbit IgG secondary antibodies (Molecular Probes, Eugene, OR, USA) in 1% BSA in 1 × PBS occurred during 1 hr. The slides were mounted with Vectashield containing DAPI (Vector Laboratories, Burlingame, CA, USA) and cells were examined using a Leica fluorescent microscope (where exposure time and gain settings were set according to the background levels of the secondary antibody only sample).

Cells were incubated overnight at 4°C with anti-human Nucleostemin (R&D Systems, Minneapolis, MN, USA; AF1638, 10 μg/ml). Cells and primary antibody were incubated with a 1:200 dilution of Northern Lights™ anti-goat IgG (NL493) for 1 hour in the dark. Cells were washed and incubated with DAPI as above. Confocal microscopy was performed using an Olympus FV1000-MPE Confocal/Multiphoton Microscope; absorption 493 nm, emission 514 nm.

For organotypic cultures, frozen sections were permeabilized with 80% MeOH for 5 min. at 4°C and acetone for 2 min. at −20°C prior to staining. Nonspecific staining was blocked by 10% goat serum. Sections were incubated for one hour in a PBS solution containing one of the following primary antibodies: Keratin-10 (1:200, clone DE-K10; Thermo Scientific, Waltham, MA, USA), E-Cadherin (1:400, clone EP700Y; Epitomics, Burlingame, CA, USA), Involucrin (1:200, clone SY5; Sigma-Aldrich, St. Louis, MO, USA), Keratin-14 (1:200; Thermo Scientific, Waltham, MA, USA), or p63 (1:100, clone EPP5701; Abcam, Cambridge, MA, USA). Primary antibodies were visualized by staining one hour with goat anti-mouse rhodamine red or goat anti-rabbit Alexa 488 (Molecular Probes, Invitrogen, Carlsbad, CA, USA). Nuclei were counterstained with 30-minute incubation with Hoechst stain (1:2000; Invitrogen Carlsbad, CA, USA). Double-staining for keratin-14 and involucrin was described previously [23]. EdU was visualized using a kit as per manufacturer’s instructions (Click-It, C10337; Invitrogen). Stained slides were viewed and photographed using an IX-71 inverted fluorescence microscope with DP71 camera and cellSens software (Olympus Imaging America Inc, Center Valley, PA, USA).

The negative controls of all immunofluorescence were isotype antibodies used at the identical concentration.

### Clonality

100 μL of K-HME 496 cell suspension (10 cells/ml WIT-P) was added to each well of two Primaria 96 well plates and two collagen-coated 96 well plates (BD Biosciences, San Jose, CA, USA). Cells were observed at day 1 of culture to identify wells with one cell only present and then subsequently observed for the growth of colonies from the single cell.

### Telomerase activity by the Telomeric Repeat Amplification Protocol (TRAP)

Telomerase activity was measured using the TRAP-eze Telomerase Detection kit (Billerica, MA, USA) and established protocols [25,26].

### Flow cytometry

Flow cytometry was carried out using a FACSCalibur flow cytometer (BD Biosciences, San Jose, CA, USA) and the CellQuest program to capture at least 10,000 events.

### Quantitative PCR

Human embryonic stem cell total RNA was obtained from Celprogen (San Pedro, CA, USA). Total RNA was isolated from the K-HME cells using the miRNeasy® Mini Kit (Qiagen, Valencia, CA, USA). PCR amplification was carried out using a BioRad CFX96 Real-Time System C1000 (BioRad, Hercules, CA, USA). cDNA was synthesized using the Tetro cDNA Synthesis Kit (Bioline, Tauton, MA, USA). 100 ng of cDNA mixed with 5 μL 2× SensiMix SYBR N0-Rox Kit (Bioline) and 200 nM of each primer (Sigma, St. Louis, MO, USA; Applied Biosystems, Carlsbad, CA, USA: *OCT4* (*POU5F1*) primer/probe set, Hs04260367_gH, cat# 4351372, *NANOG* primer/probe set Hs04260366_g1, cat# 4351372; and *GAPDH* primer/probe set, Hs99999905_m1, cat# 4331182) in a final volume of 10 μL.

## Results

### K-HME cells express common epithelial and myoepithelial markers by immunohistochemistry

**Figure 2:**
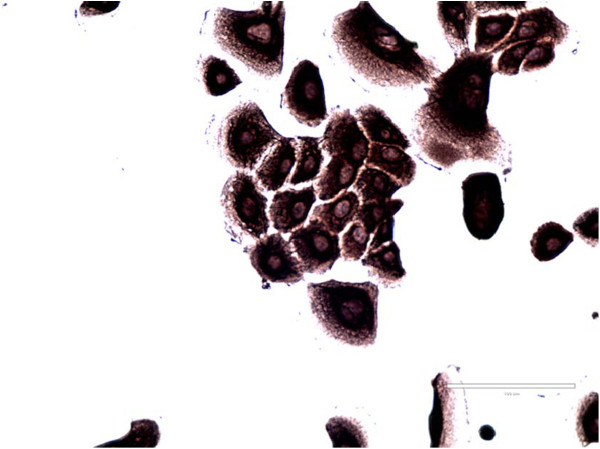
**Verification of epithelial lineage.** Immunohistochemistry using the AE1/AE3 antibody mixture in representative K-HME cells. AE1 recognizes acidic (type 1) cytokeratins (K10, 15, 16 and 19) and AE3 all known basic (type II) cytokeratins.

The K-HME cells are epithelial in nature (AE1/AE3 positive; Figure 2) and do not express estrogen receptor (ER), progesterone receptor (PR) or HER2 protein (Table 1). Cytokeratin 5/6 expression was observed in cells cultured in both MEGM and WIT-P media; cytokeratin 8/18 expression was seen only when grown in WIT-P. The expression of other proteins varied as a function of the growth media; for example vimentin expression was observed in WIT-P media but not in MEGM (Table 1). The epithelial cells expressed p63, a protein essential for the self-renewal of stem cells of epithelia [27], and a marker of myoepithelial cells [28]; and alpha smooth muscle actin, which is associated with both myoepithelial cells and myofibroblasts.

**Table 1:** Characterization of the cells by protein expression. Immunohistochemistry and immunofluorescence were performed, and expression of various markers evaluated as a function of growth surface and media

**Media: MEGM;**		**Media: Wit-P**	
**Surface: Plastic**		**Surface: Primaria**	
**Marker**	**Cells**	**Marker**	**Cells**
ER	-	ER	-
PR	-	PR	-
HER2	-	HER2	-
CK 5/6	+	CK 5/6	+
p63	+	p63	+
SMA	+/−	SMA	+
vimentin	-	vimentin	+
CK 19	-	CK 19	-
EGFR	-	CK 14	+
		CK 8/18	+
		AE 1/3	+

### The majority of K-HME cells are diploid

**Figure 3:**
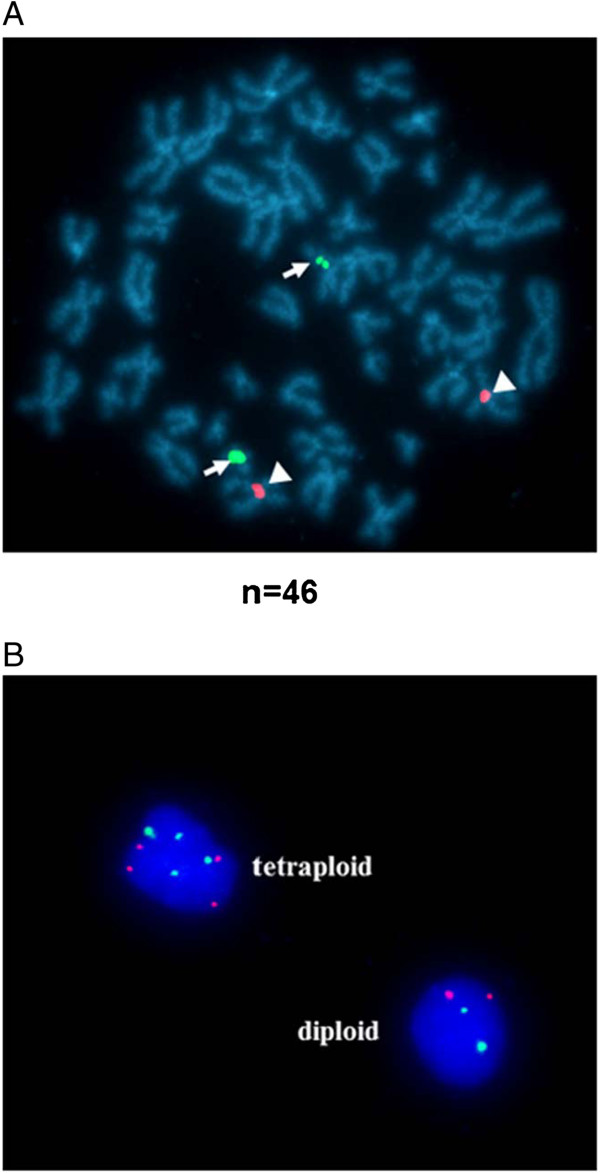
**Ploidy status in K-HME cells. A**. Metaphase chromosome spread hybridized with X (red; arrowheads) and 17 (green; arrows) centromere probes. **B**. Interphase cells hybridized with X (red) and 17 (green) centromere probes. The majority (96.9%) of the established breast epithelial cells are diploid (190/196 cells) as evidenced by two chromosome 17 and two X chromosome signals. A small percentage of cells (3.1%) were tetraploid at passage 3 and exhibited four X chromosome and four chromosome 17 signals.

The majority of the established breast epithelial cells are diploid (96.9%; 190/196 cells) as evidenced by two chromosome 17 and two chromosome X signals. A small percentage of cells (3.1%) were tetraploid at passage 3 and exhibited four X signals and four chromosome 17 signals each (Figure 3).

### Cell phenotype of the K-HME cells in the presence of basement membrane proteins

**Figure 4:**
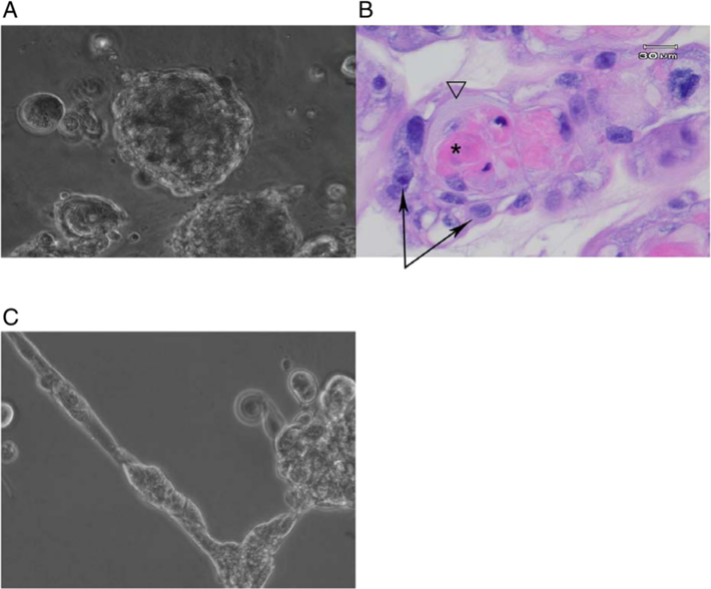
**Squamous differentiation.** K-HME cells were grown in the center of a Matrigel® sandwich. **A**. Phase contrast; and **B**. Hematoxylin and eosin stained section after 10 days. Bright-pink center is keratin (*), the triangle indicates an area equivalent to the stratum corneum, the arrows identify cells equivalent to the stratum basale of a stratified epithelium. **C**. Phase contrast of similar culture after 1 day 20×

When the cells were placed in the center of a sandwich of Matrigel®, they uniformly formed spheres 37 mm-325 mm in diameter after 10 days in culture (Figure 4A). Hematoxylin and eosin staining of the formalin-fixed and paraffin-embedded sections of these spheres reveals a keratinizing squamous epithelium (Figure 4B). The cells initially appeared to be forming duct-like structures, which disappeared over the 10 days of culture (Figure 4C).

Immunohistochemistry was repeated on sections showing squamous differentiation. In comparison to cells in 2-D culture, these cells strongly expressed the epidermal growth factor receptor and vimentin; did not express SMA (Additional file 3: Figure S1A,C,E) and contained a few, scattered CD10 positive cells (Additional file 3: Figure S1D). Single cells appeared CK18 positive; however, those cells within the spheres were very weakly CK 18 positive; ER, PR, HER2 remained negative.

**Figure 5:**
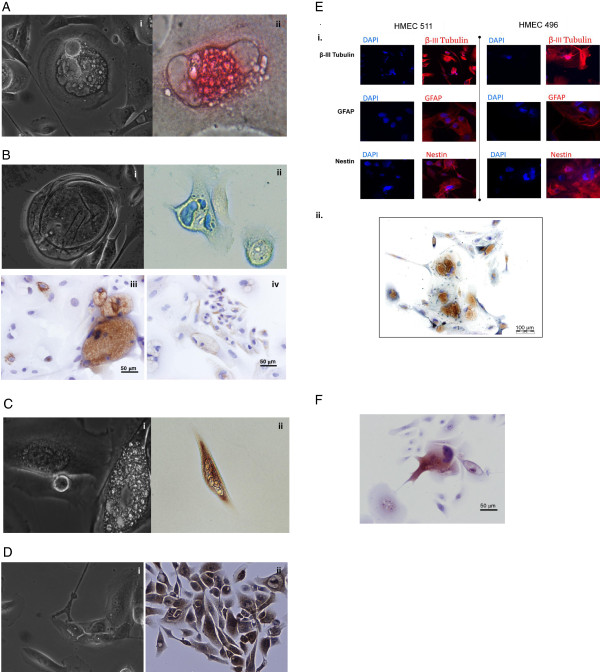
**Differentiation of K-HME cells.** Osteoclast: **A**.i. Phase contrast, Laminin, 20x, A.ii. Tartrate resistant acid phosphatase (TRAP). The bright pink is indicative of TRAP activity. Chondrocyte: **B**.i. Phase contrast, 40x, ii. Alcian blue staining. The blue color is due to the glycosaminoglycans synthesized by chondrocytes. iii. IHC anti-collagen II, iv. IHC anti-collagen X. Adipocyte: **C**.i. Phase contrast, ii. Oil Red O staining. Muscle: **D**.i. Phase contrast, Collagen IV, 20x; ii. MyoD immunohistochemistry. MyoD is an immature muscle marker. Neural: **E**.i. immunofluorescence β-III tubulin, glial fibrillary acid protein (GFAP) and nestin. β-III tubulin is expressed by differentiated neurons. GFAP is expressed by mature astrocytes and distinguishes astrocytes from other glial cells during development. Nestin expression marks stem cells of the central nervous system. ii. IHC anti-NeuN. Melanocyte: **F**. Immunohistochemistry using anti-MART-1 (anti-Melan-A; melanocyte differentiation antigen), 20x. Positive and negative controls stained appropriately, **A-F**.

Three of the twelve K-HME cell strains (K-HME 538, K-HME 496, and K-HME 511) were chosen for additional characterization. Photomicrographs of the breast tissue from two of these three donors are presented in Additional file 3: Figure S2A,B. These cells were grown on Collagen I, Collagen IV, Laminin, Fibronectin and Primaria surfaces. The most striking differentiation was observed on the Collagen IV and Fibronectin surfaces, although this was observed on Primaria dishes as well. Multiple cell types were observed including large, multinucleated cells, which resembled osteoclasts both by morphology and upon Tartrate Resistant Acid Phosphatase staining (Figure 5Ai, ii). Incubation of the cell cultures with Alcian blue highlighted the presence of glycosaminoglycans in another subfraction of the cells (Figure 5Bi, ii). Immunohistochemistry using antibodies directed against collagen II and X revealed granular cytoplasmic staining in a fraction of cells when grown on collagen or Primaria (Figure 5Biii and iv, respectively). The phenotype of the cells, the positive staining with Alcian blue, and the production of collagen II and X suggest chondrocytic differentiation [29,30].

Spindle-shape cells with Oil Red O positive cytoplasmic vacuoles consistent with adipocytes were observed (Figure 5Ci, ii). In other areas of the cell culture, a sheet of MyoD expressing cells was seen (Figure 5Di, ii). Numerous cells with long, dendritic processes expressed nestin, glial fibrillary acidic protein (GFAP), and beta-III tubulin (Figure 5Ei). Immunohistochemistry using antibodies directed at the neuron-specific nuclear protein NeuN (or Neuronal Nuclei) [31] showed uniform nuclear staining (Figure 5Eii) in a fraction of cells when grown on collagen but not on Primaria. A subpopulation of cells expressed the melanocyte differentiation antigen MART-1 (Figure 5F). The addition of BMP4 or neuregulin to the culture media did not enhance osseous or neural differentiation, respectively. Growth of the cells in Melanocyte Growth Medium did not promote the expression of MART-1.

### Clonality

Analysis of a single cell suspension of K-HME 496 cells revealed that the various cell types are represented in a colony that grew from a single cell (Additional file 3: Figure S3). Each cell type represented only a fraction of the total cells (Additional file 3: Figure S4A). Flow cytometric analysis determined that 25% of cells expressed CD151 (chondrocyte) [32,33] and 5% expressed the Calcitonin R (osteoclast) [34] (Additional file 3: Figure S4B).

### Organotypic culture

**Figure 6:**
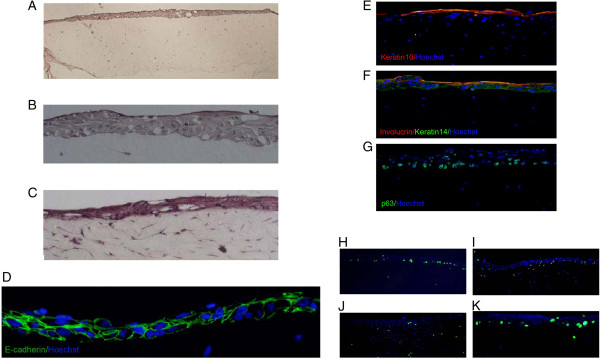
**Organotypic cultures.** K-HME cells form a stratified epithelium in organotypic cultures. **A**.**B**. Culture after 1 day, emerged; **C**. culture after 7 days, emerged. **D**. E-cadherin staining. **E**. Keratin 10, a suprabasal keratin of the epidermis. **F**. Involucrin (red) found in the upper strata of the epidermis and Keratin 14 (green) a basal-layer keratin. **G**. p63 found in the basal layer of the epidermis. **H-K**. Cultures were treated with ethynyl deoxyuridine (EdU) followed by a ‘click’ reaction to visualize cells that had entered S phase (green). **H**. Day 1 emerged; **I**. Day 3 emerged; **J**. Day 7 emerged; **K**. Day 3 emerged, magnified.

In organotypic culture, K-HME cells partially differentiated into a stratified epithelium that showed E-cadherin rich junctions characteristic of skin (Figure 6A-D). Keratin 10, a suprabasal keratin of the epidermis, was also noted (Figure 6E). Similarly, expression of involucrin (Figure 6F), a crosslinking protein found in upper strata of epidermis and Keratin-14 (Figure 6F), a basal-layer keratin of epidermis, were observed in the top and bottom layers, respectively. p63, which is expressed in the basal layer of epidermis, also was present in the K-HME cells at the bottom of the tissue (Figure 6G). There was no change in staining after 1, 3 and 7 days of emersion. Cell division occurred in cells near the basal layer with approximately 10% of the basal cells undergoing cell division (Figure 6H-K).

### Expression of breast stem cell markers in K-HME cells

**Figure 7:**
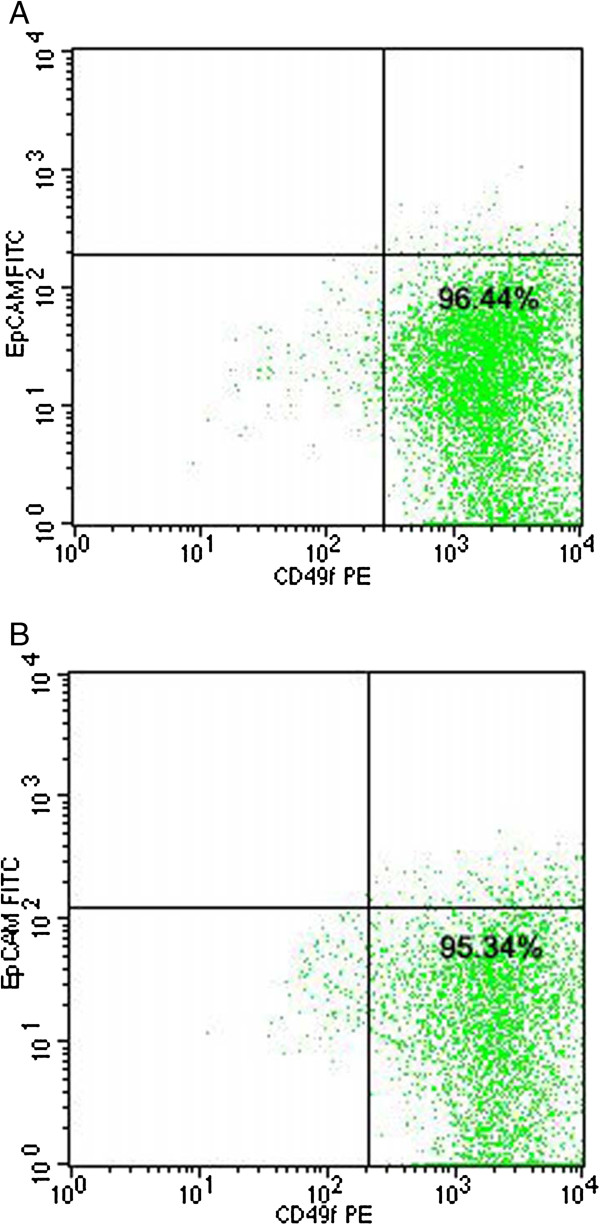
**Flow cytometry.** Representative FACS dotplots of K-HME cells labeled with CD49f and EpCAM antibodies. The CD49f^hi^EpCAM^−^ subset has been associated with the mammary stem cell enriched subpopulation [47]. **A**. K-HME 496; **B**. K-HME 511.

**Figure 8:**
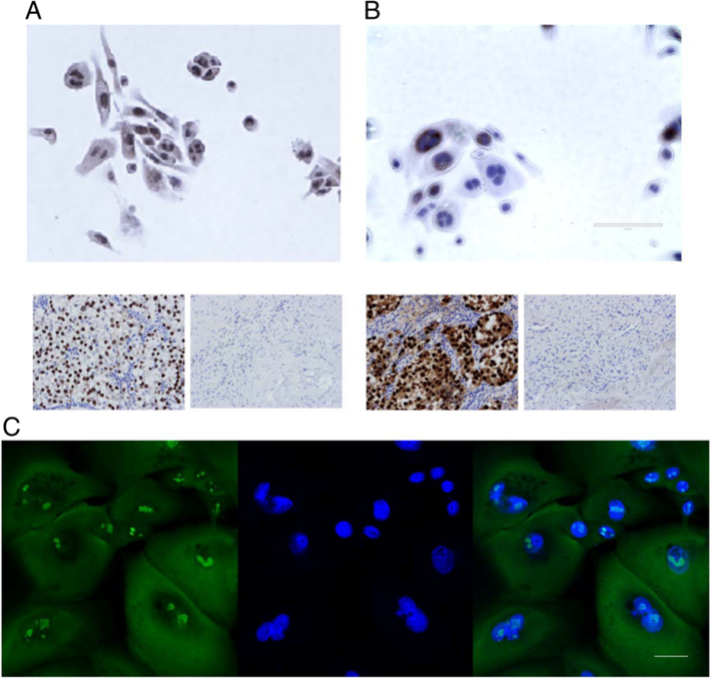
**Embryonic stem cell markers.** Immunohistochemistry: **A**. NANOG; **B**. OCT4. The majority of nuclei in the NANOG IHC are brown indicating the presence of NANOG. Some, but not all, nuclei are positive for OCT4. Negative (secondary antibody only) and positive (seminoma) controls are presented in the thumbnails below the photomicrographs. **C**. Immunofluorescence against nucleostemin. Left: anti-nucleostemin antibody, center: DAPI, right: merge. Bar = 10 microns.

Flow cytometry for stem cells markers showed the cells were CD49f positive and EpCAM negative (Figure 7). K-HME cells grown in WIT-P media were positive for telomerase activity, an indicator of progenitor cell characteristics, while those grown in MEGM were negative unless transduced with exogenous hTERT. (Additional file 3: Figure S5) Quantitative PCR revealed that the K-HME cells express the stem makers *KLF4*, *NANOG*, *NOTCH1* and *SOX2* at levels similar to embryonic stem cells (Additional file 3: Figure S6). Data is normalized to GAPDH. Expression of embryonic stem cell markers NANOG and OCT4 was verified at the protein level and was observed in a fraction of the cells by immunohistochemistry (Figure 8A, B). *OCT4* RNA expression was lower by qPCR, which is consistent with the IHC as not all cells expressed the protein. Immunofluorescence using antibodies against nucleostemin confirmed the presence of multiple nucleoli in a subset of cells (Figure 8C).

## Discussion

Identification of the mammary epithelial stem cell has been a “source of much contention” [35]. Methodologies utilized for the identification include mammosphere culture, fluorescence-activated cell sorting, and recapitulation of the mammary gland by single cells *in vivo*. “Since metaplasia often involves the transformation of undifferentiated stem or progenitor cells…” [36], metaplastic ability may be another attribute of these cells.

While no single marker can be considered to be cell-type specific, the preponderance of evidence presented in this paper including cellular phenotype, colorimetric reactions and multiple immunostains suggest that the K-HME cells are multipotent. Explant culture conditions select cells that are multipotent. Multipotency has been demonstrated for explant cultures of the hair follicle, bronchiole and intestine [10-14]. Sieber-Blum and colleagues showed that cells from bulge explants of whiskers of transgenic mice are pluripotent, differentiating into neurons, smooth muscle, Schwann cell, melanocytes, and chondrocytes [10]. Using Wnt10cre/R26R double transgenic mice, they were able to trace these cells to the neural crest. A subsequent study by Yu *et al.* confirmed differentiation into neurons, muscle cells, endothelial cell, adipocytes and osteoblasts [11]. The cell line developed by Delgado and colleagues from bronchiole explants co-express differentiation markers for multiple cell types of the lung and give rise to all lung epithelial lineages [12]. Intestinal explant cultures or “organoids” are multi-cellular aggregates of intestinal mucosal progenitors and putative mucosal stem cells, which have been seeded onto scaffolds in tissue engineering experiments to create neointestines [13,14]. The cells most competent to emerge from tissue explant cultures would appear to be the basal cells, which display the highest level of potency [12]. Similarly, the epithelial cells described herein established by outgrowth from explant culture are basal and retain multipotency. Both Delgado *et al.* and Yu *et al.* suggest that their isolated cells resemble multipotent embryonic progenitors either in terms multi-lineage differentiation and/or expression of NANOG and OCT4. Human lung stem cells isolated by Wang and colleagues express NANOG, OCT3/4, SOX2 and KLF4 [37]. Indeed, expression of OCT4 and NANOG has been reported in rare cells within adult tissues including bone marrow, epidermis, bronchial epithelium, myocardium, pancreas and testes [38]. Likewise a subpopulation of K-HME cells express OCT4 and NANOG. K-HME cells also display multiple nucleoli, a characteristic of human embryonic stem cells [39].

A recent publication from the laboratory of Tlsty and colleagues reports findings similar to the ones presented in this manuscript [40]. The cells utilized for their studies were isolated by a completely different method, i.e., the selection by flow cytometry of cells from reduction mammoplasties that, after lineage depletion, are CD73 positive and CD90 negative. These cells are also pluripotent and express a number of genes reported to confer multi-and pluipotency at levels comparable to embryonic stem cells. Although there are a number of similarities to the K-HMEs there are also some differences, e.g., their cells are EpCAM positive, and differentiation was effected by the addition of growth factors and supplements to the media. These differences notwithstanding, the fact that two independent laboratories using different methods have identified pluripotent, plastic cells in the breast lends credence to this discovery.

The epithelial cells described herein are metaplastic. They express basal cytokeratins 5 and 14, which are the hallmarks of the basal cells of stratified squamous epithelia [3], and myoepithelial cells/basal cells of the normal breast. However, a subset of luminal cells in the terminal ducts also express cytokeratin 5 [35]. In the mouse mammary gland, the basal cell fraction is enriched in mammary stem cells [41]. The expression of the luminal cytokeratins 8 and 18, and of vimentin in WIT-P media is of interest. WIT-P media in contrast to MEGM contains all-trans retinoic acid (ATRA), which has been shown to significantly increase the expression of cytokeratins 8, 18, 19, vimentin and ICAM-1 in oral gingival cells *in vitro*[42]. It also increases expression of these cytokeratins in T47D breast cancer cells [43]. Ince, Weinberg and colleagues, selected primary breast epithelial cells by their growth in WIT-P media and transfected them with hTERT, SV-40 LT/st and H-ras-v12. The xenograft tumors formed from these cells expressed cytokeratins 8 and 18 and resembled human invasive ductal carcinoma [44]. Those cells selected by their growth in MEGM resulted in tumors with squamous differentiation that lacked CK8/18 expression. p63, which is routinely used as a marker of myoepithelial cells, is strongly expressed by the K-HME cells. However, it is also a stem cell marker in the epidermis and limbal epithelium [45]. In p63-null mice, the epithelium fails to stratify, and mammary buds or other epidermal appendages do not form [46]. Pellegrini and colleagues have argued that the phenotype of p63-null mice should be ascribed to a failure to maintain the stem cell compartment. This would suggest that p63 marks the stem cells of the epidermal appendages, which includes the mammary glands, as well as the epidermis and limbic epithelium. It is entirely possible that the K-HME cells are the p63, CK14 and nestin positive cells identified by Li *et al.* in the basal/myoepithelial layer of the mammary gland [15]. K-HME cells are EpCAM negative and CD49f positive by FACS analysis, an immunophenotype ascribed by Lim *et al.* to the mammary stem cell enriched population [47].

The differentiation of human breast cells obtained from outgrowth of organoids into squames is well described [48]. The ability of these basal cells to form “relatively large spherical structures with a central core of squamous metaplasia” on basement membrane has also been noted [49,50]. Squamous differentiation of cells isolated from reduction mammoplasty has more recently been reported [51,52]. Both nasal airway stem cells and tracheal airway stem cells form spheres of squamous cells “akin to squamous cell metaplasia” when grown on Matrigel® [53]. It should be noted that the squamous differentiation observed in our study is contextual: In the middle of Matrigel®, the phenotype most closely represents a squamous carcinoma of the skin. This keratin pearl-like structure is the form assumed by squamous metaplasia in the breast both in benign (e.g. adenomyoepitheliomas) or malignant (e.g., metaplastic squamous cell carcinoma) lesions. It is also observed in squamous cell carcinoma of the lung, esophagus, anus and even in a minority of tibial adamantinomas [54]. This suggests a commonality in the pathophysiology of the metaplasia, that is, that basal/stem cells on becoming surrounded on all sides by basement membrane/stroma form keratin pearl structures. The fact that this is observed in normal cells raises the possibility that metaplasia is a property of all epithelia, which is kept in check by the normal microenvironment and tissue polarity. In a study conducted by Miyoshi and colleagues using transgenic mice, stabilization of β-catenin expression through MMTV-Cre-induced deletion of exon 3 results in reversion to epidermis and squamous metaplasia in the mammary tumors that develop therein [55]. This squamous metaplasia resembles that seen in the Matrigel® sandwich cultures in that there is a cyst-like/nodular structure with keratin in the middle encircled by a stratified epithelium. These investigators suggest that the differentiation of the mammary gland as a secretory epithelium requires suppression of β-catenin signaling, and absent this repression the phenotype reverts to epidermis [55]. In other words, the default genetic program for epithelial cells in the breast may be epidermis and their differentiation into a gland requires, at a minimum, the repression of the default program, if not a concomitant activation of a program that results in gland formation.

How can differentiation into these various cell types be explained? Boecker and colleagues recently published a study of salivary gland tumors of the breast and histologically similar tumors of the salivary and lacrimal glands [56]. They utilized triple immunofluorescence to trace the lineage of cells within these tumors. The results of their study led them to hypothesize that there are K5/K14/p63-positive progenitor cells within these neoplasms that give rise to glandular epithelial cells, myoepithelial cells, as well as the squamous and mesenchymal cells. The K-HME cells may be the progenitor cells hypothesized by Boecker *et al.*

Eric Neilson has suggested that terminal differentiation rather than being an end point is a lay-over point: “…terminal differentiation is really just an evolutionary pause maintained by signaling events, transcription factors, and genomic setting” [57]. Neilson and his colleague, Michael Zeisberg, have proposed that epithelial plasticity is comprised of two processes: Metaplasia (transdifferentiation) and epithelial-mesenchymal transition (EMT) [57,58]. EMT can further be divided into three types [58,59]. Type 1 EMT functions in early embryogenesis when it is involved in gastrulation and neural crest migration. Type 2 EMT is the formation of fibroblasts from secondary epithelial cells or endothelial cells. Type 3 EMT facilitates the metastasis of epithelial cells in a process that includes the loss of intercellular connections, migration and the establishment of residence in a secondary location.

Metaplasia is often composed of the tissue type normally derived from the neighboring region of the embryo [36,60]. A dividing line forms between these two regions at a point where an inducer is at its threshold concentration [61]. If a stem cell originally residing in this region and now in one of the resulting adult tissues retains bidirectional tissue potential, an inciting event after birth, e.g., infection, wounding, tissue regeneration, could tip the balance resulting in an homeotic transformation. The cells that eventually form the breast begin their life as ectoderm, which borders the neural crest. Neural crest cells form cartilage, bone, nerve and smooth muscle in face and cranium; as well as the peripheral nervous system and a number of neuroendocrine cell types. Pleomorphic adenomas, tumors that also display areas of bone and cartilage formation, are hypothesized to have a contribution from neural crest cells based upon the expression of GFAP [62]. Human genetic disorders may provide an additional clue. Mutation of p63 is responsible for Limb-mammary syndrome (OMIM #603543), the features of which include hypoplasia/aplasia of the mammary gland and cleft palate. That the phenotype is manifest in tissues derived from the ectoderm and neural crest suggests that the mutation was present in a progenitor of both lineages. Are the K-HMEs just such a cell? If so, the observed phenotypic plasticity observed in the K-HME cells may be more akin to Type 1 rather than Type 3 EMT.

## Conclusions

Within the normal human breast are epithelial cells with phenotypic plasticity. They are likely the source of metaplasia. Metaplasia may offer a wealth of clues with regard to normal and pathologic physiology. The human body has regulated differentiation so that specialized tissues and cells are generated and located/arranged to enable the organism to survive, thrive and function. When this differentiation goes awry, it should prompt the question: Why? What is a chondrocyte doing in the breast? Many tissues have been shown to contain cells that are pluri/multipotent. These cells function in the maintenance of tissue homeostasis or the restoration of tissue integrity following wounding or remodeling. The plasticity of the K-HME cells mimics that seen in MCB and it is tempting to postulate that these tumors arise from similar multipotent or plastic cells. The differentiation repertoire of these cells may be circumscribed under normal physiologic conditions by the tissue microenvironment. Alteration of the microenvironment by a mechanical and/or disease processes may release the restrictions enabling the metaplastic phenotype to become evident.

## Abbreviations

2-D: Two-dimensional; °C: Degree centigrade; APC: Allophycocyanin; ATRA: All-trans retinoic acid; BB: Blocking buffer; BMP4: Bone morphogenetic protein 4; bp: Base pair; BSA: Bovine serum albumin; cat.: Catalog; CK: Cytokeratin; DAPI: 4′,6-diamidino-2-phenylindole; EdU: 5-ethynyl-2′-deoxyuridine; EMA: Epithelial membrane antigen; ER: Estrogen receptor; FACS: Fluorescence activated cell sorting; FITC: Fluorescein isothiocyanate; FISH: Fluorescence in situ hybridization; g: Gravity; GFAP: Glial fibrillary acidic protein; HER2: Human Epidermal Growth Factor Receptor 2; HBSS: Hank’s Balanced Salt Solution; HI: Heat inactivated; HME: Human mammary epithelial; hTERT: Human telomerase reverse transcriptase; IC: Internal standard control; ICAM-1: Intercellular adhesion molecule 1; IgG: Immunoglobulin G; IHC: Immunohistochemistry; IRB: Institutional review board; IUH: Indiana University Health; IUSCC: Indiana University Melvin and Bren Simon Cancer Center; KTB: Susan G. Komen for the Cure® Tissue Bank at the IU Simon Cancer Center; LB: Lysis Buffer; LT: Lifetime; MCB: Metaplastic carcinoma of the breast; MEGM: Mammary epithelial growth medium; MeOH: Methanol; MMTV: Mouse mammary tumor virus; MSC: Mammary stem cell; NA: Not applicable; NIH: National Institutes of Health; no.: Number; OMIM: Online Mendelian Inheritance in Man; PBS: Phosphate buffered saline; PCR: Polymerase chain reaction; PE: Phycoerythrin; PR: Progesterone receptor; R: Receptor; RT: Room temperature; SMA: Smooth muscle actin; TRAP: Tartrate resistant acid phosphatase; WB: Wash buffer.

## Competing interests

The authors have no competing interests to disclose.

## Authors’ contributions

BH established the cells. SEC and BH conceived and designed the study and analyzed the data. CAMS, JEK, MC, MJF, RJB, MR, BH, and SEC performed experiments and analyses; BRG performed the FISH and ploidy analysis. MBV and CCL carried out the organotypic culture experiments. SB provided expertise in Pathology. SEC, BH, SB, MBV wrote and revised the manuscript. All co-authors edited the manuscript. All authors read and approved the final draft of this manuscript.

## Supplementary Material

Additional file 1: Table S1 Age, ethnicity and Gail breast cancer risk estimates of tissue donors. **Table S2.** Immunohistochemistry protocols. Click here for file

Additional file 2 Methods. Click here for file

Additional file 3: Figure S1 Immunohistochemistry of the cells in Matrigel® sandwiches showing squamous differentiation. **Figure S2.** Hematoxylin and eosin stained sections of breast tissue adjacent to the core utilized for the production of epithelial and stromal cells. **Figure S3.** Phase contrast photomicrograph of cell colony grown from a single cell (Evos x1, Advanced Microscopy Group, Bothell, WA; 40x objective). **Figure S4.** A. Tartrate-resistant acid phosphatase staining of K-HME 511 cells grown on laminin (40x). Positive staining cells are in the minority. B. FACS analysis of K-HME 511 cells. PE = anti CD151; ACP = anti-Calcitonin R(recptor). **Figure S5.** Telomerase activity of K-HME and K-HMS cells grown in different media (WIT-P, DMEM/F12, and MEGM) using the polymerase chain reaction (PCR)-based TRAP-eze assay. **Figure S6.** Graphical representation of quantitative PCR results. Click here for file
