# Supplementary material for: Phenotypic plasticity in normal breast derived epithelial cells
Source: BMC Cell Biol. 2014 Jun 10;15:20. doi: 10.1186/1471-2121-15-20 (PMC4066279; doi:10.1186/1471-2121-15-20)
Supplement: Additional file 1: Table S1 — Age, ethnicity and Gail breast cancer risk estimates of tissue donors. Table S2. Immunohistochemistry protocols. [file 1471-2121-15-20-S1.doc]

| **Sample ID#** | **Age** | **Ethnicity** | **Gail Score (5yr/LT)** | **Epithelial Cells*** | **Stromal Cells*** |
| --- | --- | --- | --- | --- | --- |
| 102488 | 46 | W | 0.8/9.3 | + |  |
| 102489 | 37 | AA | 0.4/9.8 | + | + |
| 102490 | 37 | W | 0.4/11.2 | + |  |
| 102491 | 37 | W | 0.8/19 | + | + |
| 102492 | 37 | W | 0.4/11.2 | + | + |
| 102493 | 52 | W | 1.2/9.6 | + | + |
| 102494 | 39 | W | 0.6/11.1 | + |  |
| 102496 | 25 | W | NA | + | + |
| 102504 | 39 | W | 0.6/12.2 | + | + |
| 102505 | 62 | W | 3.2/14 | + | + |
| 102506 | 63 | W | 1.9/8.1 | + | + |
| 102507 | 26 | W | NA |  | + |
| 102509 | 30 | AA | NA | + | + |
| 102510 | 49 | AA | 1.1/8.8 |  | + |
| 102511 | 31 | AA | NA | + | + |
| 102513 | 46 | W | 1.1/11.8 | + | + |
| 102516 | 50 | W | 2.2/19.2 | + | + |
| 102517 | 50 | W | 0.9/8.0 | + | + |
| 102518 | 39 | W | 0.6/11.1 |  | + |
| 102520 | 43 | W | 1.4/18.3 | + | + |
| 102521 | 63 | W | 3.6/14.5 |  | + |
| 102522 | 40 | AA | 0.4/7.5 |  | + |
| 102524 | 61 | AA | NA | + | + |
| 102525 | 55 | W | 1.8/12.2 | + | + |
| 102528 | 44 | AA | NA | + | + |
| 102531 | 38 | AA | 0.5/9.7 | + | + |
| 102535 | 32 | W | NA | + | + |
| 102536 | 29 | W | NA | + | + |
| 102537 | 59 | W | 3.6/18.4 | + | + |
| 102538 | 72 | W | 5/12.6 | + | + |
| 102539 | 20 | W | NA |  | + |
| 102540 | 57 | AA | 2.2/11.7 | + | + |
| 102541 | 23 | W | NA | + | + |
| 102546 | 40 | AA | 0.6/9.6 |  | + |
| 104255 | 50 | W | 9.9 | + | + |
| 104257 | 33 | W | NA | + |  |
| 104260 | 27 | W | NA | + | + |
| 104269 | 66 | W | 11 |  | + |
| 104273 | 30 | W | NA | + | + |

Additional file 1: Table S1.

Table S2.

| Antibody | p63  (Dako, Carpinteria, CA, USA)  1:400 | SMA 1A4  (Dako)  RTU | CD10  (Dako)  RTU | Vimentin  (Dako)  RTU | OCT 4  (Cell Marque, Rocklin, CA, USA)  RTU | NANOG  (Abcam, Cambridge, MA, USA)  1:100 | MART1  also known as Melan A (Dako)  RTU | MyoD1 (Dako)  1:50 |
| --- | --- | --- | --- | --- | --- | --- | --- | --- |
| Antigen retrieval | Flex High pH (Dako) in PT Module (Dako)  20 min | Flex High pH in PT Module 20min | Flex High pH in PT Module 20min | Flex Low pH (Dako) in PT Module 20min | Flex High pH in PT Module 20min | Flex Low pH in PT Module 20min | Flex High pH in PT Module 20min | Flex High pH in PT Module 20min |
| 3% H_2_O_2_ | 5min,  rinse TBS |  |  |  | 10 min,  rinse TBS |  | 5min,  rinse TBS |  |
| Protein Block |  |  |  |  |  | 15 min,  tap off |  |  |
| Primary Ab | 10 min,  rinse TBS | 10 min,  rinse TBS | 10min,  rinse TBS | 10 min,  rinse TBS | 10min,  rinse TBS | 20 min,  rinse TBS | 10min,  rinse TBS | 20 min,  rinse TBS |
| Flex Linker | Flex  + Mouse Linker (Dako) 10min,  rinse TBS |  | Flex  + Mouse Linker  10min,  rinse TBS |  | Flex  + Mouse  Linker  10min,  rinse TBS | Flex  + Rabbit Linker  (Dako) 10min,  rinse TBS | Flex +Mouse Linker 10min,  rinse TBS | Flex +Mouse Linker  15min,  rinse TBS |
| Flex HRP | 10min, rinse TBS |  |  |  |  |  |  | 15 min, rinse TBS |
| Flex DAB | Flex DAB 10min, rinse TBS |  |  |  |  |  | Nova Red (Novacastra, Leica, Buffalo Grove, IL, USA)  20min | Flex DAB 10min, rinse TBS |
| Flex Hema-toxylin | Flex Hema-toxylin 7min,  rinse H_2_O |  |  |  |  |  |  |  |
| Dehydrate, clear, coverslip |  |  |  |  |  |  |  |  |

| Antibody | Neu-N  (Chemicon, Billerica, MA, USA )  1:50 | Collagen II  (Abcam, Cambridge, MA, USA)  1:200 | Collagen X (Abcam, Cambridge, MA, USA)  1:2000 |
| --- | --- | --- | --- |
| Antigen retrieval | Flex High pH in PT Module (Dako)  20 min | Flex Low pH in PT Module (Dako) 20min | Flex Low pH in PT Module (Dako) 20min |
| 3% H_2_O_2_ | 5min,  rinse TBS |  |  |
| Protein Block | x | x | x |
| Primary Ab | 20 min,  rinse TBS | 10 min,  rinse TBS | 40 min,  rinse TBS |
| Flex Linker | x | x | x |
| Flex HRP | 10min, rinse TBS |  |  |
| Flex DAB | Flex DAB  2 x 5min, rinse TBS |  |  |
| Flex Hema-toxylin | Flex Hema-toxylin 7min,  rinse H_2_O |  |  |
| Dehydrate, clear, coverslip |  |  |  |

Table S1. Age, ethnicity and Gail breast cancer risk estimates of tissue donors. “+” indicates cells successfully established and cryopreserved. AA=African-American, W=White.

Table S2. Protocol for immunohistochemistry using the Dako AutostainerPlus (Carpinteria, CA, USA).
