# Supplementary material for: Phenotypic plasticity in normal breast derived epithelial cells
Source: BMC Cell Biol. 2014 Jun 10;15:20. doi: 10.1186/1471-2121-15-20 (PMC4066279; doi:10.1186/1471-2121-15-20)
Supplement: Additional file 2 — Methods. [file 1471-2121-15-20-S2.doc]

Additional file 2: Methods

*Cell culture*

After digestion, the remaining tissues, termed organoids, were separated from the rest of the digested material using 60-100 µm filters and centrifugation (80 x g, 1-5 min). To isolate epithelial cells, the organoids were placed on typical cell culture plastic dishes in Medium-171 (Cascade Biologics/Invitrogen, Carlsbad, CA, USA) supplemented with 0.4% bovine pituitary extract (Hammond Cell Tech, Windsor, CA, USA), 10 ng/ml epidermal growth factor (Invitrogen, Carlsbad, CA, USA), 5 μg/ml insulin, 0.5 μg/ml hydrocortisone, 5 μg/ml transferrin [14-17]. This supplemented media was hereafter referred to as MEGM. Reagents were obtained from Sigma (St. Louis, MO, USA) unless otherwise noted. Aliquots of isolated cells were cryopreserved for future use. Replicate, established cultures could also be grown on Primaria™ dishes (BD Falcon, San Jose, CA, USA) in defined WIT-P medium (Stemgent, Cambridge, MA, USA) [41]. The filtrate was collected and plated in media containing DMEM/F12 supplemented with 15% FBS plus 5 μg/ml each insulin and hydrocortisone (Sigma) as a source of matched stromal cells from the same individual [17].

*3-D Matrigel® culture*

Matrigel® (BD Bioscience, San Jose, CA, USA) was removed from the freezer, placed on ice and stored in the refrigerator the evening prior to initiating cultures. Once the cells were at least 70% confluent in the T-75 flasks, they were trypsinized, collected in media and counted using a hemocytometer. 500 µl of Matrigel® (at 4°C) were used to cover the bottom of the wells of a 24-well plate. Plates were placed in the incubator (37°C) for 30 minutes to enable the Matrigel® to semi-solidify. The cell suspension was diluted to final concentration of 1000 cells/µl using WIT-P. 125 µl of the suspension was pipetted onto the surface of the Matrigel® and the plates were incubated for 5 minutes at room temperature (RT) to enable the cells to adhere to the Matrigel®. Media was then aspirated. 500 µl of Matrigel® (4°C) were pipetted onto the cells and the sandwich culture was incubated for 30 minutes at 37°C. 1-2 ml of WIT-P was then added to the cultures, which were returned to the incubator. The media was changed three times per week. After approximately 10 days, the sandwich cultures were placed in Histogel (Richard-Allan Scientific, Kalamazoo, MI, USA) as follows. Cold packs, normally utilized in insulated shipping containers, were frozen and then wrapped with paper towels. Histology tissue cassettes were placed on top of the wrapped ice packs. Histogel was placed in a boiling water bath for approximately 10 minutes or until completely liquefied and then cooled at RT. It was then pipetted into the cooled cassettes where it solidified forming a floor. The sandwich cultures were removed from the wells of the 24-well plate using a spatula and placed into the center of the cassette on top of the solidified Histogel. Histogel was then added to cover the sandwich culture and the Histogel embedded cultures were cooled until solidified. The cassettes were then placed in 10% buffered formalin (Fisher Scientific, Pittsburgh, PA, USA) and transferred to Indiana University Health (IUH) Pathology Laboratory for routine paraffin-embedding and sectioning. Sections were stained with hematoxylin and eosin, and unstained sections were provided for immunohistochemistry.

*Differentiation analyses of cultures grown on coated plates*

***Chondrocytic differentiation***

# Alcian blue (Alcian Blue pH 2.5 Stain Kit, Artisan™, Dako, Carpinteria, CA, USA). Cells were fixed using 3% glutaraldehyde for 15 minutes at RT and then rinsed with 0.1N hydrochloric acid (HCl). 1 ml of 1% Alcian blue was added to the well and allowed to react at RT for 30 minutes. Excess stain was removed by rinsing twice with 0.1N HCl. Cells were allowed to dry for 5 minutes and then rehydrated with 0.1N HCl before visualization.

**2 wells of 4 well chamber slides (catalog number 154526, Lab-Tek, Scotts Valley, CA) were coated with a 3 mg/mL collagen solution (StemCell Technologies, Vancouver, BC, Canada). Collagen solution was added to completely cover the bottom of the slide. Following overnight incubation at 37°C, the remaining collagen solution was removed, slides were washed once with 1X HBSS, and stored at 4 degrees prior to use. Collagen II and X immunohistochemistry performed as given in Additional file 1, Table 2.**

# *Adipocytic differentiation*

Oil Red O (Sigma-Aldrich, St. Louis, MO, USA). Following fixation, cells were rinsed with nuclease free water three times, five minutes each. After incubation with Oil Red O, the cells were washed three times with nuclease free water, 1 hour each wash before visualization.

Phase contrast images were obtained using a Nikon TS 100-F inverted microscope (Nikon, Melville, NY, USA) fitted with a SPOT Insight Fire Wire Camera (Diagnostic Instruments, Inc.; Sterling Heights, MI, USA).

***Melanocytic differentiation***

The concentration of the collagen solution was 3mg/mL. The coated plates were incubated at 37ºC overnight. Excess collagen was removed and the wells were rinsed with HBSS. After fixation, wells were washed with HBSS and after the final wash HBSS+1%BSA was added to the wells.

*Neural differentiation*

Slides were incubated with the collagen solution for 1 hour at RT. Any remaining solution was aspirated. The collagen was washed three times with 1x PBS. 3 x10^4^ cells were plated per well and incubated for 4-5 days. Media was aspirated and cells were washed in 1x HBSS for 2 minutes before fixation in 4% paraformaldehyde, 0.15% picric acid in PBS for 20 minutes at RT. Cells were washed for 5 minutes in Wash Buffer (WB, 0.1% BSA (Sigma-Aldrich, St. Louis, MO, USA) in 1x PBS) at RT before blocking non-specific binding using Blocking Buffer (BB; 0.1% Triton X-100 (Sigma-Aldrich, St. Louis, MO, USA), 10% Donkey serum (Abcam, Cambridge, MA, USA), and 1% BSA (Sigma-Aldrich, St. Louis, MO, USA) in 1x PBS) for 45 minutes at RT. The cells were washed again with WB for 5 minutes. Following incubation with the primary antibody, cells were washed with WB three times, 5 minutes each. After incubation with the secondary antibody, cells were washed with WB for 5 minutes. Subsequent to DAPI, cells were washed three times with PBS, 5 minutes each. Confocal microscopy was performed using an Olympus **FV1000-MPE Confocal/Multiphoton Microscope; a**bsorption 557nm, emission 574nm. Neuroblastoma cells, used as positive controls, were a kind gift of Dr. Linda Malkas.

**2 wells of 4 well chamber slides (catalog number 154526, Lab-Tek, Scotts Valley, CA) were coated with a 3 mg/mL collagen solution (StemCell Technologies, Vancouver, BC, Canada). Collagen solution was added to completely cover the bottom of the slide. Following overnight incubation at 37°C, the remaining collagen solution was removed, slides were washed once with 1X HBSS, and stored at 4 degrees prior to use. Neu-N immunohistochemistry performed as given in Additional File 1, Table 2.**

*Immunofluorescence*

Following incubation with anti-human Nucleostemin, cells were washed with WB for 5 minutes x 3.

*Quantitative PCR*

PCR cycles consisted of an initial step at 95 °C for 10 min, 50 cycles at 95 °C for 10 sec and 58 °C for 30 sec, followed by 72 °C for 15 sec, and the melt curves were generated at 65-95 °C at 0.5 increments for 5 seconds. Data analysis was performed using the software provided with the thermal cycler; expression was normalized to GAPDH.

*Clonality*

K-HME 496 cells were grown on Primaria coated flasks until 70% confluent. Cells were then trypsinized, washed and resuspended in WIT-P media. They were counted using a hemocytometer.

*Flow Cytometry*

K-HME cells were collected at 70% confluency and resuspended in WIT-P containing 10% FBS. The cells were counted and then divided equally among five 1.5 ml Eppendorf tubes. Following centrifugation for 5 minutes at 1500 x g, media was aspirated and the pellet resuspended in 500 µl of Flow Wash Buffer (FWB; 0.02% sodium azide, 0.5% BSA in 1x PBS). The suspension was centrifuged 5 min at 1500 x g and the media aspirated. The pellet was resuspended in 200 µl FWB containing 2 µg/ml human IgG (Innovative Research, Novi, MI, USA; IR-HU-GF) and incubated for 30 min at 4º C. Eppendorf tubes 1-5 were treated as follows: #1: cells only; #2: 20 µl of IgG1 FITC Isotype (isotype control mouse IgG 1kappa monoclonal-FITC conjugated antibody, abcam, Cambridge, MA, USA; AB 18435) and 20 µl of IgG2a PE Isotype (BD Biosciences, San Jose, CA, USA; 555844); #3: 40 µl FITC-conjugated EpCAM antibody (clone VU-19D; StemCell Technologies, Vancouver, BC, Canada; 10109); #4: 40 µl PF-conjugated CD49f antibody (BD Biosciences, San Jose, CA, USA; 555736); #5: 40 µl of each antibody used in tubes 3 and 4. The suspensions were then incubated in the dark at 4ºC for 1 hour. They were centrifuged for 5 min at 1500 x g, the solutions aspirated and the cell pellet washed with FWB a total of three times. After the final centrifugation, the FWB was aspirated and the pellets resuspended in 300 µl of fixation solution (2% paraformaldehyde in 1x PBS). Fixation proceeded in the dark for 1 hour at 4º C. The cells and fixation solution were centrifuged at 1500 x g for 5 min., the solution aspirated, and the cells resuspended in 500 µl of 1x PBS before transferring to separate flow cytometry tubes.

65 x 10^4^ K-HME 511 cells were plated in each well of seven 6-well Collagen IV Coated Plates (BD Biosciences, San Jose, CA, USA; 354428). The media was changed every other day for 1 week. All wells were then washed with HBSS. Cell Dissociation Buffer (Life Technologies, Carlsbad, CA, USA; 13150016) was added for 1-2 hours until cells lifted off bottom of the wells. Cells were then counted and separated into 5 tubes (cells only, isotype controls, anti-CD151, anti-Calcitonin R, anti-CD151+ anti-Calcitonin R). Cells were centrifuged at 1280 rpm for 10 minutes and resuspended in FcR blocking buffer (90µL PBS+1%BSA + 10µL FcR reagent). After 10 minute incubation at 4ºC, the cells were centrifuged for 10 minutes at 1280 rpm and resuspended in HBSS. Subsequently, 1µL of reconstituted LIVE/DEAD stain (Invitrogen, Carlsbad, CA, USA; L34955) was added to each tube, except the cells only, and incubated on ice for 30 minutes. The cells were then washed with HBSS and centrifuged at 5000 rpm for 10 minutes at 4ºC. Cells were resuspended in 100µL of antibody cocktail (90µL PBS+1% BSA+ 10µL antibody, or 80µL PBS+ 1% BSA +10µL each antibody for combined tube). After 15 minutes incubation at 4ºC, cells were washed twice with 1mL HBSS and resuspended in 1% paraformaldehyde. Cells were then stored at 4ºC for 48 hours until flow. The day the flow analysis was completed, Comp beads (BDCompBeads, BD Biosciences, San Jose, CA, USA; 552843) were prepared as controls for the experiment. Beads were prepared with LIVE/DEAD stain and antibodies were used according to manufacturer’s instructions.
