# Supplementary material for: Phenotypic plasticity in normal breast derived epithelial cells
Source: BMC Cell Biol. 2014 Jun 10;15:20. doi: 10.1186/1471-2121-15-20 (PMC4066279; doi:10.1186/1471-2121-15-20)
Supplement: Additional file 3: Figure S1 — Immunohistochemistry of the cells in Matrigel® sandwiches showing squamous differentiation. Figure S2. Hematoxylin and eosin stained sections of breast tissue adjacent to the core utilized for the production of epithelial and stromal cells. Figure S3. Phase contrast photomicrograph of cell colony grown from a single cell (Evos x1, Advanced Microscopy Group, Bothell, WA; 40x objective). Figure S4. A. Tartrate-resistant acid phosphatase staining of K-HME 511 cells grown on laminin (40x). Positive staining cells are in the minority. B. FACS analysis of K-HME 511 cells. PE = anti CD151; ACP = anti-Calcitonin R(recptor). Figure S5. Telomerase activity of K-HME and K-HMS cells grown in different media (WIT-P, DMEM/F12, and MEGM) using the polymerase chain reaction (PCR)-based TRAP-eze assay. Figure S6. Graphical representation of quantitative PCR results. [file 1471-2121-15-20-S3.pdf]

S1 A.

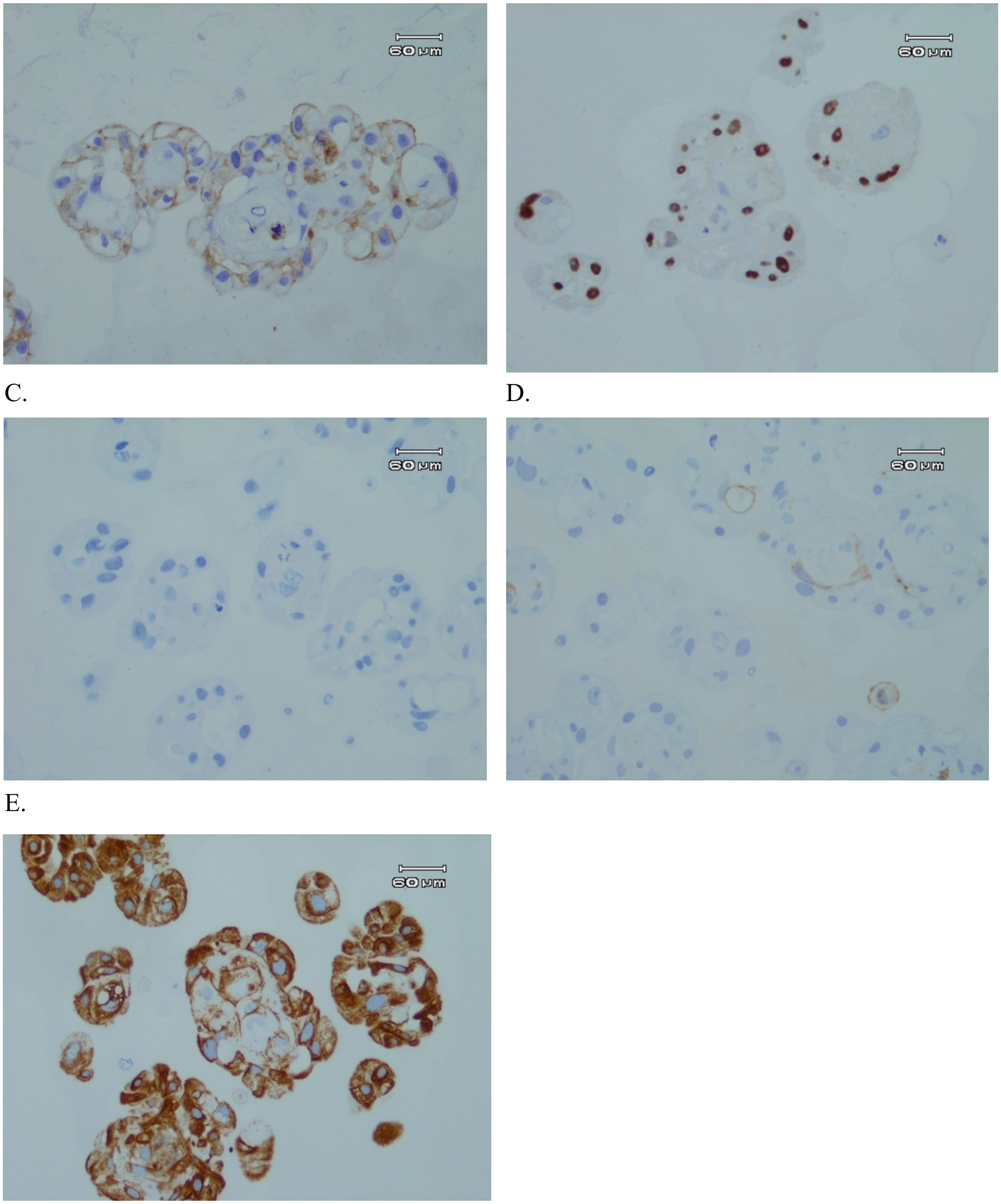

S2 A.

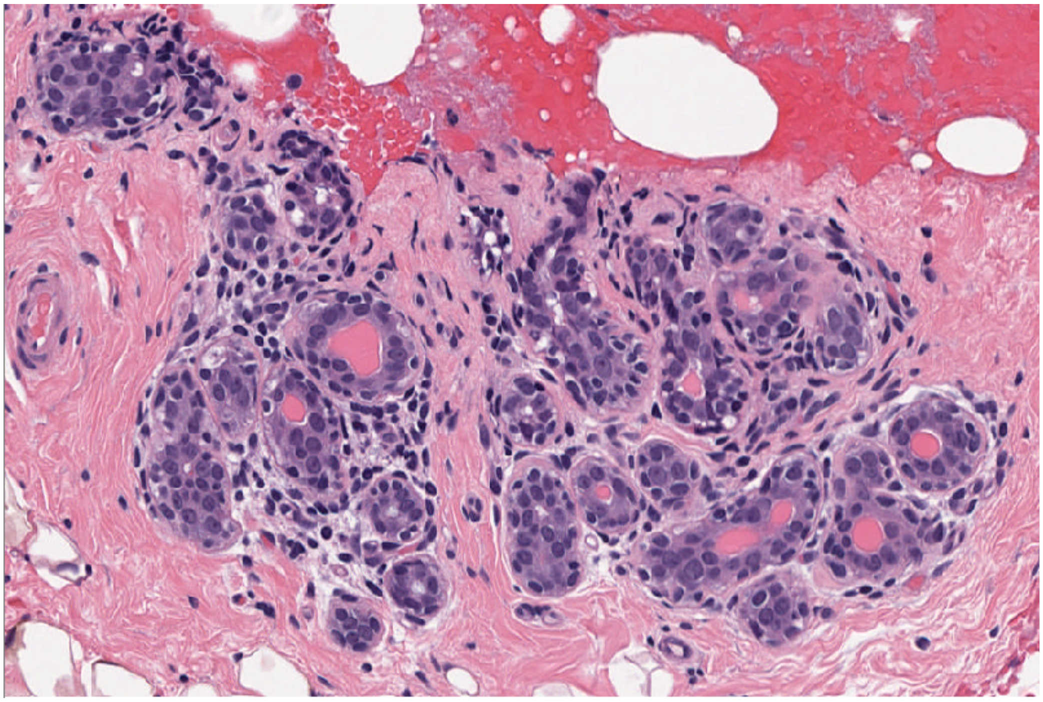

B.

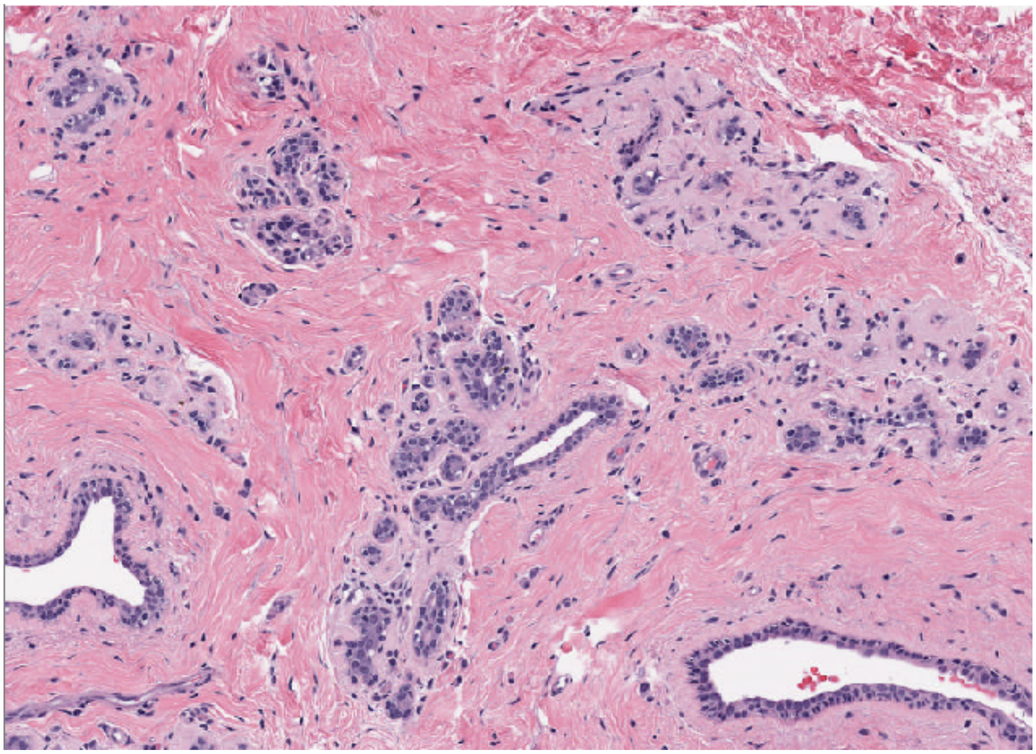

S3

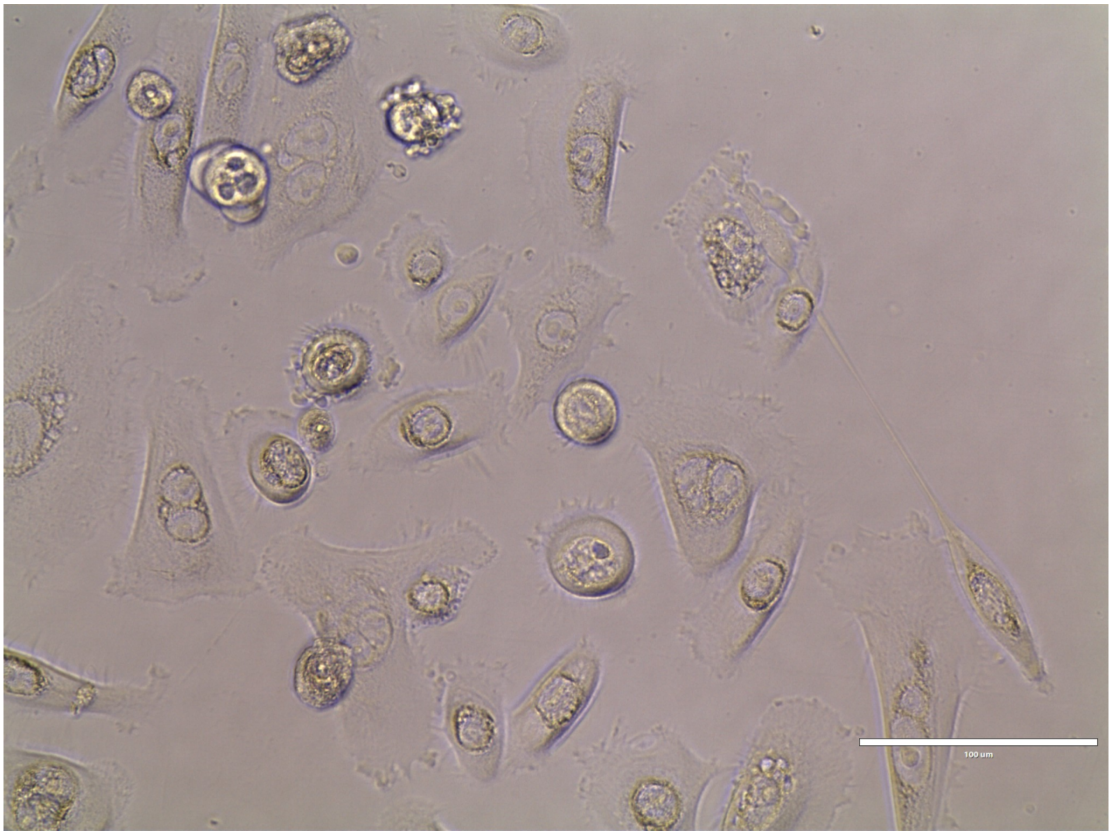

S4 A.

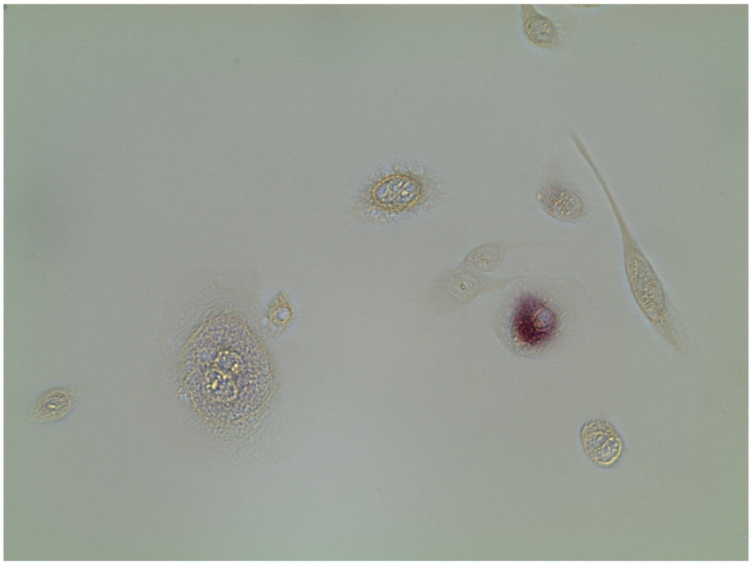

B.

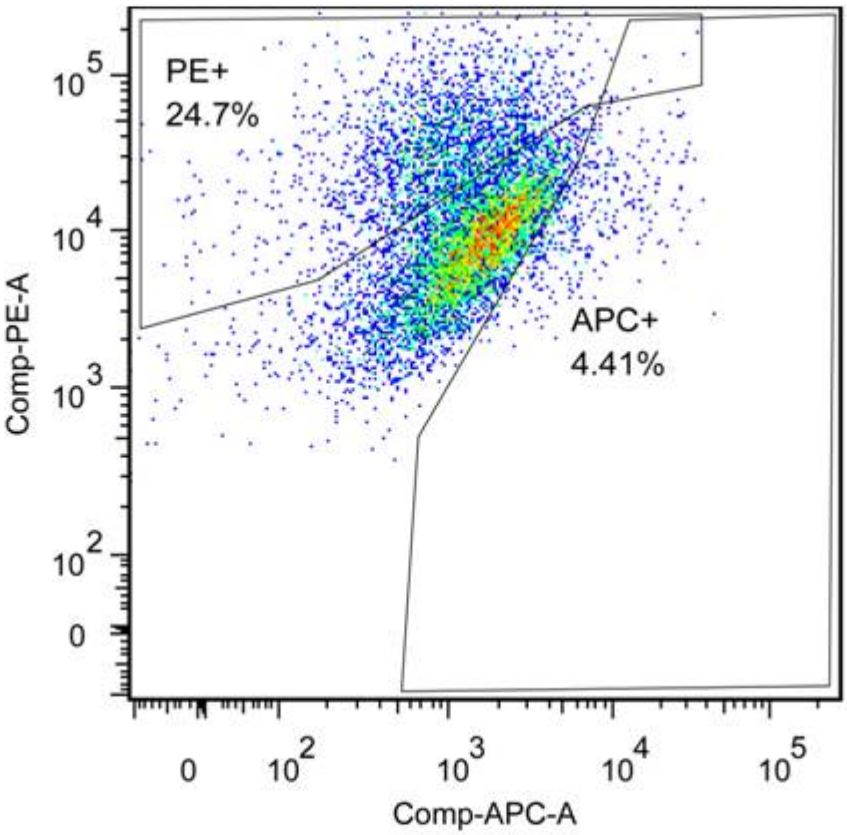
PE+
24.7%
APC+
4.41%
Comp-PE-A
Comp-APC-A
10^5
10^4
10^3
10^2
0
0
10^2
10^3
10^4
10^5

S5

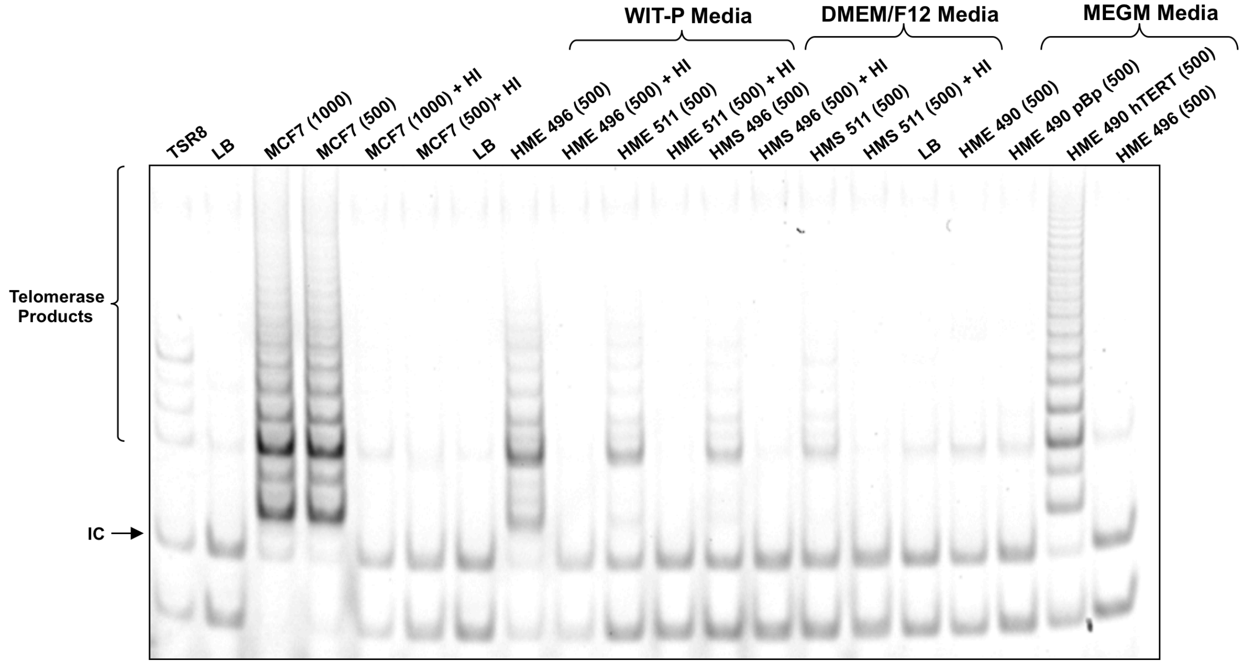
WIT-P Media
DMEM/F12 Media
MEGM Media
TSR8
LB
MCF7 (1000)
MCF7 (500)
MCF7 (1000) + HI
MCF7 (500)+ HI
LB
HME 496 (500)
HME 496 (500) + HI
HME 511 (500)
HME 511 (500) + HI
HMS 496 (500)
HMS 496 (500) + HI
HMS 511 (500)
HMS 511 (500) + HI
LB
HME 490 (500)
HME 490 pBp (500)
HME 490 hTERT (500)
HME 496 (500)
Telomerase Products
IC

S6

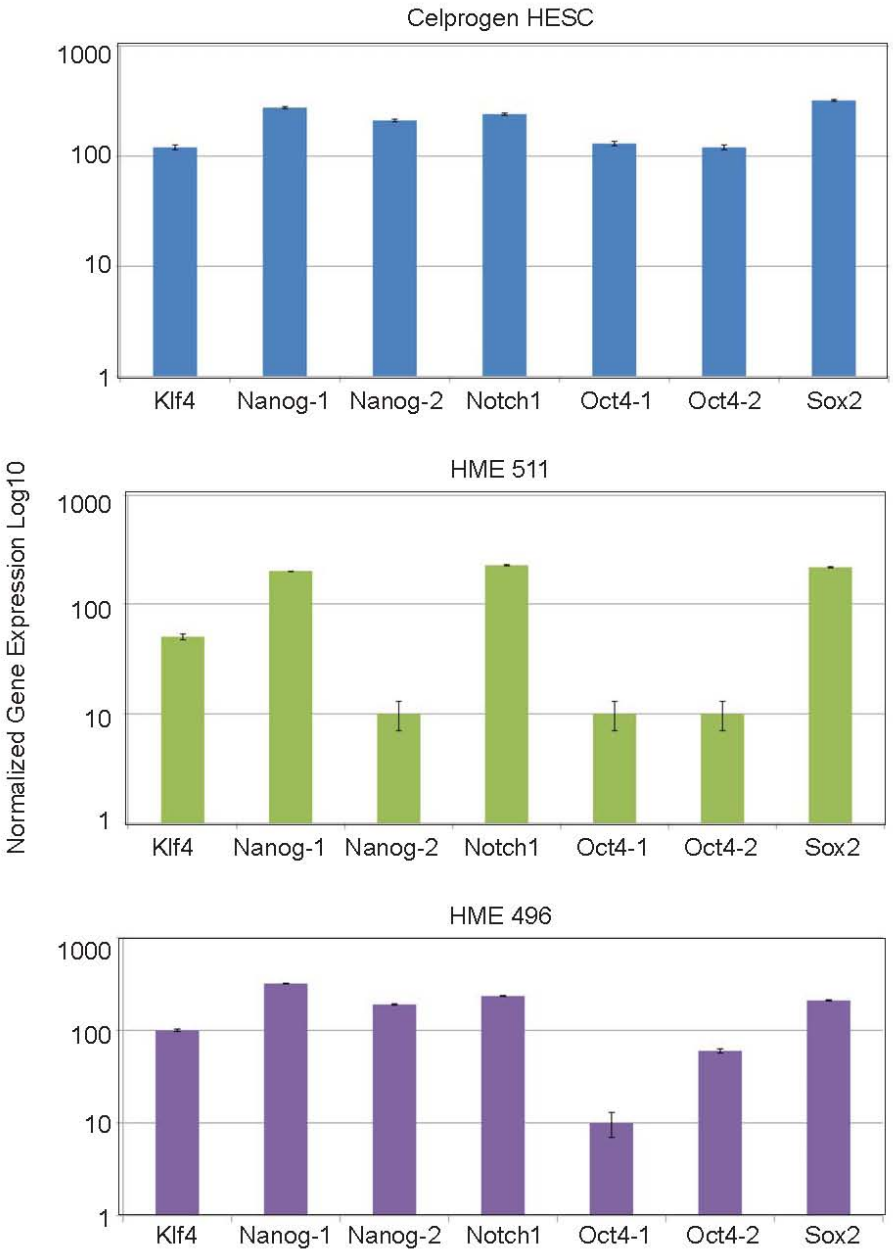

Celprogen HESC
HME 511
HME 496
Normalized Gene Expression Log10
1000
100
10
1
Klf4
Nanog-1
Nanog-2
Notch1
Oct4-1
Oct4-2
Sox2

1. Immunohistochemistry of the cells in Matrigel® sandwiches showing squamous differentiation. A. epidermal growth factor receptor; B. p63; C. smooth muscle actin; D. CD10; E. vimentin.
2. Hematoxylin and eosin stained sections of breast tissue adjacent to the core utilized for the production of epithelial and stromal cells. A. Sample ID# 102496, 10x. B. Sample ID# 102511, 4x.
3. Phase contrast photomicrograph of cell colony grown from a single cell (Evos x1, Advanced Microscopy Group, Bothell, WA; 40x objective).
4. A. Tartrate-resistant acid phosphatase staining of K-HME 511 cells grown on laminin (40x). Positive staining cells are in the minority. B. FACS analysis of K-HME 511 cells. PE = anti CD151; ACP = anti-Calcitonin R(recptor).
5. Telomerase activity of K-HME and K-HMS cells grown in different media (WIT-P, DMEM/F12, and MEGM) using the polymerase chain reaction (PCR)-based TRAP-eze assay. Cy5-labeled PCR reaction products were resolved on a 10% polyacrylamide gel and visualized with a PhosphorImager. A ladder of bands represents the extension of the substrate primer by telomerase (telomerase products). Numbers in parentheses represent cell equivalents assayed for telomerase activity. IC, internal amplification standard control (36 bp). Lysis buffer (LB) alone heat inactivated (HI) samples served as a negative controls. TSR8 control template oligonucleotide and MCF7 carcinoma cells served as positive controls for the assay. The telomerase activity levels of K-HME 490 cells grown in MEGM medium were compared to K-HME 490 cells transduced with hTERT or empty vector control (pBabepuro, pBp).
6. Graphical representation of quantitative PCR results. HESC = Human Embyonic Stem Cells, HME 511 = K-HME 511, HME 496 = K-HME 496. Scale is logarithmic. NANOG-2 and OCT4-2 primers were purchased from Applied Biosystems, all other were obtained from Sigma.
